# A report on the Global Open Water Swimming (GOWS) Conference, Cork, Ireland, 12th October 2013

**DOI:** 10.1186/2046-7648-2-34

**Published:** 2013-12-01

**Authors:** Heather Lunt, Sakura Hingley

**Affiliations:** 1 Extreme Environments Laboratory, Department of Sport and Exercise Science, University of Portsmouth, Portsmouth PO1 2ER, UK; 2 Ealing Hospital NHS Trust, Uxbridge Road, Southall, Middlesex UB1 3HW, England

**Keywords:** Marathon swimming, Cold water swimming, Open water swimming

## Abstract

The third Global Open Water Swimming Conference was for the first time held in Europe. Researchers, swim pioneers and leading marathon swimmers came together to present research related to the water environment, pioneer swims, young and old marathon swimmers and human physiology.

## GOWS 2013

The Global Open Water Swimming Conference 2013 invited novice and experienced open water swimmers from all corners of the world to congregate in Cork, Ireland to discuss open water swimming. The conference was opened by the Mayor of the County of Cork. The opening session commenced with a description of the technical, physical and emotional difficulties in completing the 'Oceans 7 Swimming Challenge’. This challenge consists of the successful completion of seven channel swims: the English Channel (UK), Catalina Channel (USA), the North Channel (UK), the Cook Straits (NZL), the Molokai Channel (HI, USA), the Tsugaru Channel (Japan) and the Straits of Gibraltar (Spain). Stephen Redmond (IRE), the first swimmer to complete the Oceans 7 Challenge, and Anna-Carin Nordin (SWE), the second successful swimmer and first female to complete the Challenge conducted the presentation; it was followed by a question and answer session.

Following on from this, Dr. Tom Doyle (University College Cork, Ireland) discussed the abundance of jellyfish in the UK and Irish coastal waters, their temporal patterning and aggregation in light of the open water swimming season. Prof. Angel Yanagihara (University of Hawaii, HI, USA) presented her recent research in box jellyfish and treatment of stings. Yanagihara has studied the variation of movement of the box jellyfish based on different coloured lights and time of day. Of particular interest was the information on the micro-anatomy of the box jellyfish, the mechanisms of its venomous sting and the development of anti-venom, which Yanagihara has developed in her laboratories, having personally experienced the excruciating consequences of a box jellyfish sting during her deep-sea diving experiences. Prof. Robert Devoy (University of Cork, Ireland) concluded the opening session discussing the impact of climate change and global warming and predictions on how this will impact on sea currents, surface water temperatures and elevation of sea levels in the next century and beyond.

A central focus of the conference was on the wide range of ages that participate in open water swimming, including young children swimming in the sea to seasoned masters swimmers who have participated in the sport for decades. Sally Minty-Gravett (President of the Jersey Long Distance Swimming Club) presented her work on building and maintaining a successful open water swimming club, having shown huge success in supporting and developing the sport from a grass roots level in Jersey, UK. Nicholas Adams (President of the Channel Swimming and Piloting Federation) explained the variation in minimum age criteria of swimmers in different marathon swims around the world, using examples of how long-distance open water swimming age limits compare to other sports such as marathon running or triathlons. Ellery McGowan (South London Swimming Club and swimming coach of Charterhouse School) gave a short presentation describing how older swimmers can be involved in the sport by giving examples of training regimens and cross training which maintains fitness and enables continued participation in open water swimming events in old age. She has recently successfully completed marathon swims such as the Manhattan Island Marathon swim and the Ederle Swim, NYC. Niek Kloots, Dutch open water swimming promoter (co-webmaster and founder of the website 'European Open Water Swimming’) discussed how websites could promote and inform swimmers interested in participating in open water swims around Europe. The session was completed by a presentation from Kevin Murphy (King of the English Channel) on 'The Great Dual: Murphy vs. Renford’. This was a three-swim challenge between Kevin Murphy and an Australian open water swimmer, Des Renford, which made open water swimming history.

The post-lunch session focused on the physiology of exercise in cold water; we presented the past and current research into the physiology of cold water immersion, describing the cold shock response, habituation, hypothermia and non-freezing cold injury. Novel research comparing adult open water swimmers to pool swimmers and a study into children's responses to training in cold water were also presented. This was followed by Trevor Woods (University College Cork, Ireland) who discussed the science of recovery from training, how this could be improved and techniques to reduce the risk of overtraining, especially focusing on extreme training for endurance swimming events.

Highlights of the conference were a number of presentations on pioneering endurance swims, the technical issues of organising a 'new’ open water swim and the difficulties encountered, from arranging support vessels and crew, securing permission to undertake the swim by local authorities and the challenges of longer and/or colder swims. Presentations included 'firsts’ such as the following:

• A new route to cross the North Channel from the Mull of Kintyre (Wayne Soutter)

• The 5ive Island Swim Challenge, a series of circumnavigation swims around the Islands of Dragonera, Portsea, Jersey, Isle of Wight, Tiree (Anna Wardley)

• A two-way Dublin Bay swim (Fergal Somerville)

• A 60-km endurance river swim from Fermoy to Cork (Owen O'Keeffe)

• An endurance swim of Lake Konstanz (Christof Wandratch)

The final session focused on a new craze known as 'The Ice Swimming’. Ram Barkai, founder of the International Ice Swimming Association, described the rules and regulations of swimming, a recognised 'Ice Mile’, and his personal experiences in swimming in sub 5°C waters. The conference was concluded by a presentation and a question and answer session from select members of a large 66-man relay swim of the Bering Straits which was completed between the 5th and 11th August 2013. A total distance of 134 km was swum from Cape Dezhnew, Russia to the Cape Prince of Wales, Alaska, in water temperatures between 2°C and 10°C. Detailed information on the route, logistics, planning and execution of this large multinational endeavour were presented. The final presentation gave an outline of the medical provisions required on such a long, cold swim as well as some information on the medical investigations they were able to perform on all swimmers pre and post swims. This was delivered by the Chief Medical Officer of the Bering Straits relay team, Dr. Nataluiya Fatyanova.

The conference was closed with words from Ned Dennison, an open water swimmer, coach, administrator and part of the organising committee for the 2013 Global Open Water Swimming Conference.

Dates and venue for the 2014 Open Water Swimming Conference are to be decided. Look out for details on http://www.dailynews.openwaterswimming.com or http://www.worldopenwaterswimmingassociation.com.

## Abbreviations

GOWS: Global Open Water Swimming; IRE: Ireland; NZL: New Zealand; SWE: Sweden; UK: United Kingdom; USA: United States of America.

## Competing interests

Both authors declare they have no competing interests.

## Authors' contributions

Both authors, HL and SH, wrote and read the final manuscript.

